# Pattern and correlates of out-of-pocket payment (OOP) on female sterilization in India, 1990–2014

**DOI:** 10.1186/s12905-020-0884-1

**Published:** 2020-01-22

**Authors:** Sanjay K. Mohanty, Suyash Mishra, Sayantani Chatterjee, Niranjan Saggurti

**Affiliations:** 1 Department of Fertility Studies, International Institute for Population Sciences, Mumbai, India; 2 International Institute for Population Sciences, Mumbai, India; 3 Population Council, New Delhi, India

**Keywords:** Family planning, India, OOP, Compensation, Female sterilization

## Abstract

**Background:**

Large scale public investment in family welfare programme has made female sterilization a free service in public health centers in India. Besides, it also provides financial compensation to acceptors. Despite these interventions, the use of contraception from private health centers has increased over time, across states and socio-economic groups in India. Though many studies have examined trends, patterns, and determinants of female sterilization services, studies on out-of-pocket payment (OOP) and compensations on sterilisation are limited in India. This paper examines the trends and variations in out-of-pocket payment (OOP) and compensations associated with female sterilization in India.

**Methods:**

Data from the National Family Health Survey - 4, 2015–16 was used for the analyses. A composite variable based on compensation received and amount paid by users was computed and categorized into four distinct groups. Multivariate analyses were used to understand the significant predictors of OOP of female sterilization.

**Results:**

Public health centers continued to be the major providers of female sterilization services; nearly 77.8% had availed themselves of free sterilization and 61.6% had received compensation for female sterilization. About two-fifths of the women in the economically well-off state like Kerala and one-third of the women in a poor state like Bihar had paid but did not receive any compensation for female sterilization. The OOP on female sterilization varies from 70 to 79% across India. The OOP on female sterilization was significantly higher among the educated and women belonging to the higher wealth quintile linking OOP to ability to pay for better quality of care.

**Conclusion:**

Public sector investment in family planning is required to provide free or subsidized provision of family welfare services, especially to women from a poor household. Improving the quality of female sterilization services in public health centers and rationalizing the compensation may extend the reach of family planning services in India.

## Background

Investment in family planning has both short and long run returns at the societal and individual levels. At the societal level, increase in the use of family planning reduces the fertility level, stabilizes population growth in the long run and increases the level of socioeconomic development [1]. The pathways of family planning, economic growth and poverty reduction have drawn considerable attention among leading economists and demographers [2–7]. A number of studies from Asia and Africa have established the positive effect of increasing family planning use on economic growth, per capita income and reduction in poverty [8–15].

At the individual level, access to contraception increases spacing between births, reduces unintended pregnancies and pregnancy complications, reduces unmet need, helps to realise the desired family size and improves the overall health of mothers and children [3, 16–27]. Research also suggests that the use of contraception is associated with increasing household income, savings and women participation in paid employment [2, 6, 28–31]. Children of small families tend to have higher educational attainment, better cognitive development and better health [32–40]. Given the multiple benefits, many national and state governments, international donors and developmental agencies continue to support family welfare programmes worldwide [20, 41, 42]. The theoretical rationale on investment in family planning and empirical evidence on cost and benefits of family planning at both the macro and micro levels have been well established [41–44].

In 1952, India became the first country in the world to launch the centrally funded official family planning programme with the aim of reducing population growth. Since its inception, family planning services are provided free at public health centers throughout the country. Over the last six decades, the family planning programme has undergone several changes in its design, approach and execution. During the first two decades, the family planning programme had adopted a clinical approach, that is, couples who needed family planning services had to visit clinics to avail themselves of the services. To strengthen the acceptance of family planning, the ‘extension education approach’ was introduced in the 1960s, by reaching the community and educating them about the utility of small family norms [45]. In the 1970s, for a brief period of 2 years, the family planning services adopted a coercive approach and suffered severe criticism. Later, a ‘cafeteria approach’ was adopted that aimed at providing approved family planning methods in keeping with the choice of the acceptors [45]. In 1977, the family planning programme was renamed Family Welfare Programme. In the 1990s, the programme was integrated into the Maternal and Child Health Programme (MCH) and later into the Reproductive and Child Health Programme (RCH). Since 2005, the family welfare programme has been under the umbrella of the National Health Mission (NHM) that aims to address health vulnerabilities persisting in India, holistically through the life cycle approach – from infancy to adolescent to adulthood, with special focus on mothers and children [46].

A large body of literature has focused on the factors affecting female sterilization in India. The general inference from the studies suggests that female sterilization is the most used method of contraception due to convenience, free provisioning at public health centers, and provision of compensation [47–49]. The acceptance of female sterilization was higher among the poor, less educated, working women, and those who had at least one son in their family [50, 51]. Several myths about temporary methods of modern contraception restricts many women from accepting short-term reversible methods of contraception and opting for sterilization [52, 53]. A number of studies have documented the overemphasis of female sterilization in family welfare programmes, poor quality of care and limited choice of methods in both the high and low fertility states [48, 53–57].

In India, sterilization is not only provided at no cost in public health centers, but compensation is also paid to acceptors towards wage loss, transportation to and from the facility, expenses of food, child care during hospital stay and laboratory fees for related tests. The compensation amount has been revised periodically and varies in high focus states^1^ and non-high focus states and by type of provider [58]. A sum of ₹10 was provided to vasectomy acceptors in 1952 in Madras as the first case of compensation. It increased to ₹20 by 1964 and increased to ₹170 for vasectomy in 1983 [59]. In 2007, the compensation for tubectomy in public health centers was ₹600 each in high and non-high focus states, which increased to ₹1400 in high focus states and remained at ₹600 in non-high focus states during 2015–16. During this period, the amount of compensation for vasectomy was ₹1100 in both the high and non-high focus states. In 2015–16, the compensation in high focus states increased to ₹2000. Many private providers and Non-Governmental Organization (NGO) trusts under the public-private partnership programmes (PPP) provide compensation to acceptors of sterilization in India [58]. Well-designed service delivery strategies are effective in increasing the level of acceptance of contraception [59–64]. Studies suggest reconsidering the provision of compensation particularly to institutions, doctors and individual providers given India’s remarkable gains in reducing overall fertility [55].

Despite the programmatic emphasis and provision of compensation, the use of modern method of contraception has remained same or slightly declined from 48.5% in 2005–06 to 47.8% in 2015. Three–fourths of all modern contraceptive use is in the form of female sterilization in India. In terms of demographic output, the family welfare programmes in India have been successful in reducing the fertility level — a reduction in total fertility rate (TFR) from 5.2 in 1971 to 2.3 in 2016 [65]. However, regional variations in fertility levels and contraceptive use remain a concern. Modern contraceptive use was lowest in Manipur followed by Bihar and Lakshadweep and highest in Andhra Pradesh and Punjab [66].

The provisioning of free female sterilization and compensations for acceptors of female sterilization is perhaps one of the largest public investments by the national and state governments in India. While a large number of studies have examined the trends, patterns and determinants of contraceptive use with specific reference to female sterilization, there is no nationally representative study on OOP and compensation for female sterilization in India. Besides, the use of female sterilization from private health centers is on the rise across states and socio-economic groups. Findings from the fourth round of National Family Health Survey (NFHS 4) reveal that about 17% of the female sterilization users had undergone the procedure in private facilities [66]. In this context, this paper examines the inter-state variations of OOP and compensation received for voluntary female sterilization in India. We hypothesize that an increasing proportion of population is paying for female sterilization services and the inter-state variation in OOP is large in India.

## Methods

### Data

We have used unit data from the individual file of the fourth round of the National Family Health Survey (NFHS 4) conducted during 2015–16. The NFHS 4 survey had interviewed 601,509 households covering 699,686 women aged 15–49 years and 112,122 men aged 15–54 years. It has the distinction of providing district level estimates (640 districts) while the earlier rounds provided state level estimates of demographic and health variables. The results of the survey along with methodology and sampling design are available elsewhere [66]. The NFHS 4 survey data collection was conducted in two phases during January 2015 and December 2016. Data on female sterilization such as year, expenditure and compensation received for female sterilization were collected from the respondents who had undergone female sterilization. For all India analyses, we limit the cases to those who availed themselves of sterilization till 2014.

**Fig. 1:**
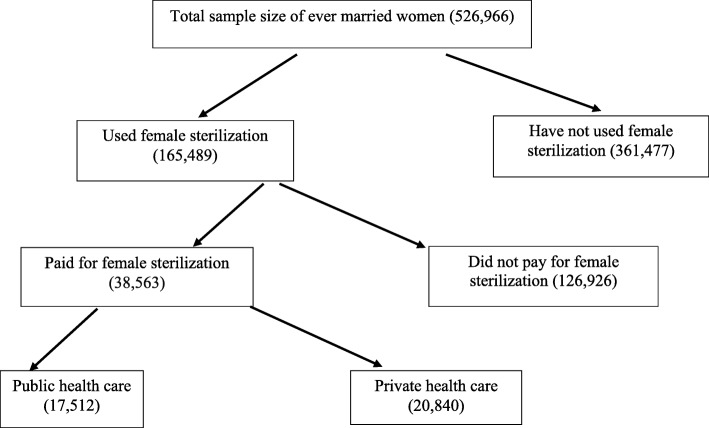
Schematic presentation of women who had accepted sterilisation by type of facility in India, 2015–16

Out of the 699,686 women interviewed, 526,966 ever married women and 172,720 were never married women in 15–49 age group. Those who were never in union, those with missing information and those who had not accepted sterilization were excluded from the analyses. The effective sample size was 165,489 women who had ever undergone female sterilization. Among them, a total of 38,561 women had paid for the sterilization services, 17,512 women were sterilized at public health centers and 20,840 availed themselves of the service from private centers (Fig. 1).

For the first time, the NFHS 4 collected information on the total amount paid for sterilization and compensation received by those sterilized. The questions pertaining to amount paid and compensation received on sterilization was

“*How much did you pay in total for the sterilization including any consultation you may have had?”,*

*“Did you receive compensation for the sterilization?”.*

*“How much compensation did you receive? .*

The data related to error for Do Not Know (DK), missing and sterilization amount paid were corrected prior to the analyses. The details and procedure of data cleaning is available elsewhere [67].

### Outcome variables

A composite variable based on compensation received and amount paid for female sterilization was used as an outcome variable. The composite variable was computed and categorized into four distinct groups:

(i) Those who neither paid nor received compensation,
(ii) Those who paid and received compensation,
(iii) Those who paid and did not receive compensation
(iv) Those who did not pay but received compensation.

The OOP is defined as the total amount spent on sterilization less compensation received.

### Independent variables

Independent variables used in the analyses includes year (time,) female sterilization by type of facility, quality of care and background characteristics. The year of sterilization is used to examine the trends in compensation received and amount paid for female sterilization. The type of facility of sterilization were broadly categorized into three; public, private and others^2^. The respondents’ perception of quality of care variable was categorized as ‘good’ (‘very good’ or ‘all right’) and ‘not good’ (‘not so good’ or ‘bad’). The other explanatory variables included in the analyses were: women’s age (less than 25, 25–34, 35–49), women’s education (no education, up to primary school, up to secondary school, high school and above), and number of surviving children. The variables relating to household included wealth quintile (poorest, poorer, middle, richer, richest), caste (scheduled caste, scheduled tribe, other backward class and others), religion (Hindu, Muslim and others), place of residence (rural and urban). Caste as a social variable and the population of India are conventionally classified into four caste groups, namely, Scheduled Caste (SC), Scheduled Tribe (ST), Other Backward Class (OBC) and others. The ST, SC and OBC are considered socially disadvantaged caste groups and the reservation for education and employment and many other benefits of national, state and local governments are made available to them.

### Statistical analysis

Descriptive analysis, adjusted OOP at constant price, bivariate analysis and two-part regression model were used in the analyses. The variations in OOP and amount of compensation received were analyzed by wealth quintile, place of residence, and educational attainment of mothers across the states of India.

The compensation received and amount paid for female sterilization was truncated at 99.5 percentile. We have presented the amount on female sterilization and compensation received between 2011 and 2016 at constant prices; based on the Consumer Price Index (CPI) available from Reserve Bank of India (RBI) for all India and for all the states. Since 2011, the CPI was computed for each states on an annual basis at 2016 prices.

### Multivariate analysis

The two-part regression method was used to understand the significant predictors of OOP and to obtain the predicted mean OOP of female sterilization in India. In a typical dataset, the outcome variable that is, OOP on female sterilization is skewed and contains a large number of zero values. In such cases, the two-part model is one of the preferred methods for analysis. The first part using logit model describes the likelihood of an individual incurring OOP on female sterilization by selected socio-demographic and economic variables.

The model takes the following form:

$$ P\left({y}_i>0\right)=\frac{\exp \beta x}{1+\exp \beta x} $$

Where y_i_ = 0 indicates that the individual has no OOP on sterilization.

The second part of the model determines the probability of a woman incurring any OOP on sterilization using Ordinary least square (OLS) regression. In the Ordinary least square (OLS) regression, the logarithm of a woman incurring any OOP on sterilization was used as a dependent variable. The model predicts the OOP on female sterilization after adjusting for selected socio-demographic and economic variables.

## Results

### Female sterilization by type of facility in states of India

**Fig. 2:**
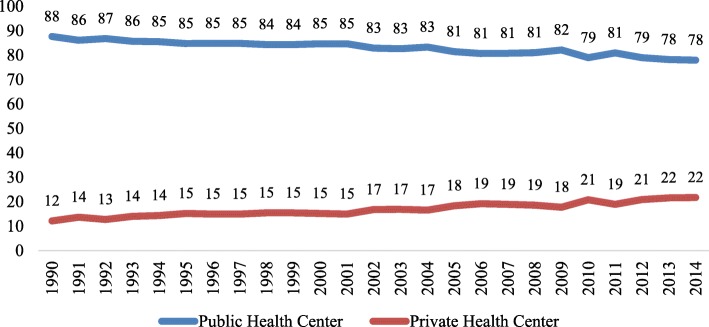
Trends in share of female sterilization (percentage) by type of facility in India, 1990–2014

Figure 2 presents the trends in female sterilization by type of facility in India. During 1990–2014, the share of female sterilization conducted in public health centers declined from 88% to 78% and that from private health centers increased from 12% to 22%. The share of female sterilization from other type of facilities remained at a low level over time (less than 0.4%). Public health centers thus remained to be the most preferred type of facility for female sterilization in India.

**Fig. 3:**
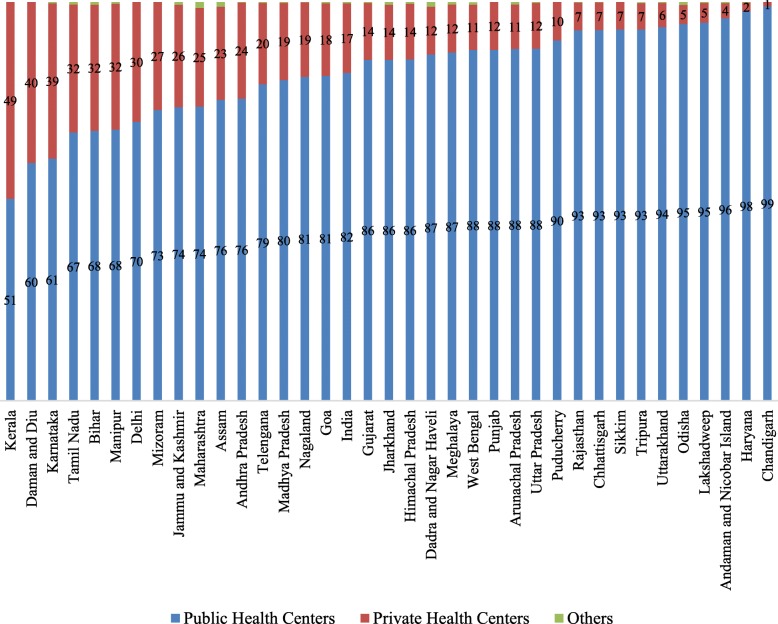
Percent distribution of female sterilization by type of facility in states of India, 2010–14

Figure 3 presents the inter-state variations in female sterilization by type of facility in India. More than 80% of the acceptors of female sterilization in India received services from public health centers. The state variations of female sterilization in public and private health centers were large. In Chandigarh, Haryana, Andaman and Nicobar Islands, Lakshadweep, Odisha, Uttarakhand, Tripura, Sikkim, Chhattisgarh, Rajasthan and Pondicherry, over 90% of the female sterilizations were carried out in public health centers. On the other hand, in Kerala, Karnataka, Telangana, Bihar, Manipur, Mizoram, Jammu and Kashmir, Delhi and Daman and Diu, about 25% of female sterilizations were carried out in private health centers. In general, most of the sterilizations were conducted in public health centers across the states of India.

### Amount paid, compensation received and OOP for female sterilization in India

Table S1 presents the percent distribution of the amount paid and compensation received for female sterilization, total expenditure (at current prices) and OOP as a share of total expenditure during 1990–2014. The majority of the women had not paid but received compensation for female sterilization (about 60%). The proportion of women who had paid for sterilisation and did not receive any compensation has almost doubled over time; from 10.2% in 1990 to 20.9% by 2014. Similar increase was also noticed for those who paid and received compensation for sterilisation. The OOP as a share of the total expenditure on female sterilization in India showed an increase from 70% to 79% with some variations over time. Table S2 presents the trends in percent distribution of the amount paid and compensation received for female sterilization, total expenditure at current price (in ₹) and OOP as a share of total expenditure by type of facility (public and private health centers) in India. Among those women who utilized female sterilization services from public health centers, the majority did not pay but received compensation. In general, over 72% women reported receiving some form of compensation from public health centers in 1990 and 85% in 2014. The majority of women availing services from private health centers had paid for the services and did not receive any compensation. It has increased from 81% in 1990 to 91% by 2014. In 1990, the mean total expenditure on sterilization was ₹97 in public health centers and ₹5406 in private health centers and ₹306 in public and ₹10,304 in private health centers in 2014 (at current price). A gradual decline in the ratio of total expenditure in private and public health centers incurred for female sterilization was observed from 2005.

Table 1 presents the distribution of composite variables based on amount paid and compensation received for female sterilization, along with the level of TFR, the OOP and its share in the total expenditure for female sterilization in the states of India during 2010–2014 at constant prices with base year 2016. At the national level, about 12% of the women had neither paid nor received compensation, 8% had paid and received compensation, 20% had paid for female sterilization but did not receive any compensation, while around 60% of the women did not pay for the service and received compensation. Among the major states, about 42% of the women in Kerala had paid but did not receive any compensation followed by Manipur and Nagaland (39%) in contrast to around 4% women in Madhya Pradesh and Himachal Pradesh respectively. In the state of Himachal Pradesh, 88% of the women did not pay but received compensation followed by Madhya Pradesh (84%); the least was found in Nagaland (23%) and Manipur (30%). The percentage of women who neither paid nor received any compensation varied from 36% in Arunachal Pradesh to 5% in Madhya Pradesh and Bihar. Nearly 46% of the women in Mizoram and 28% of the women in Bihar had paid and received compensation.

**Table 1:** Percent distribution of women who paid and received compensation, percent sterilized, TFR, mean OOP and OOP as a share to total expenditure on female sterilization by states of India at constant prices (in ₹), 2010–14

State	Neither paid nor received (%)	Paid and received compensation (%)	Paid and did not receive compensation (%)	Did not pay but received compensation (%)	TFR, 2015–2016	Percentage of women sterilized, 2015–2016	Mean OOP (₹)	OOP as a share of total expenditure on sterilization
Andaman and Nicobar Islands	25.5	1.09	5.40	68.01	1.41	39.9	671	55.3
Andhra Pradesh	13.61	1.63	27.93	56.82	1.83	68.3	3231	87.1
Arunachal Pradesh	35.93	1.47	16.62	45.97	2.26	11.21	1098	77.1
Assam	11.1	6.23	9.55	73.12	2.25	9.53	217	28.6
Bihar	5.39	27.55	28.13	38.93	3.56	20.7	1801	78.7
Chhattisgarh	7.90	3.25	6.48	82.38	2.32	46.2	242	27.2
Dadra and Nagar Haveli	18.41	3.81	4.80	72.97	2.38	31.7	97	14.8
Daman and Diu	27.59	0	44.44	27.97	1.77	25.7	6052	97.4
Delhi	13.27	8.49	25.31	52.93	1.75	19.8	2384	84.7
Gujarat	13.62	0.84	18.11	67.43	2.10	33.6	1246	73.9
Haryana	12.78	4.22	18.11	64.89	2.12	38.1	1423	80.6
Himachal Pradesh	6.52	1.60	3.99	87.90	1.91	34.5	395	39.6
Jammu and Kashmir	24.39	6.82	29.0	39.78	2.06	24.4	2992	89.2
Jharkhand	9.17	6.80	22.45	61.59	2.60	31.1	1352	70.6
Karnataka	20.39	6.79	20.57	52.25	1.79	48.6	2621	87.4
Kerala	16.9	5.48	42.05	35.57	1.56	45.8	7025	96
Madhya Pradesh	4.91	6.94	3.97	84.18	2.42	42.2	−173	−34.1
Maharashtra	20.87	3.91	25.24	49.98	1.92	50.7	2256	88.6
Manipur	25.69	5.53	38.91	29.87	2.60	3.1	7390	96.3
Meghalaya	19.33	7.08	31.5	42.09	3.20	6.2	5778	93.4
Mizoram	15.67	45.54	28.13	10.66	2.36	17.5	5343	92.8
Nagaland	20.16	17.71	38.68	23.44	2.76	9.1	8423	95.4
Odisha	13.38	12.77	8.28	65.57	2.11	28.2	311	33.9
Puducherry	21.29	2.17	11.85	64.69	1.67	57.4	1608	77.6
Punjab	22.48	2.71	14.07	60.74	1.65	37.5	895	67.1
Rajasthan	12.01	2.96	7.21	77.81	2.48	40.7	−143	−26.7
Sikkim	30.55	8.48	16.56	44.41	1.14	17.6	433	44.5
Tamil Nadu	10.35	5.46	23.87	60.32	1.76	49.4	3010	86.4
Telangana	14.09	1.78	37.48	46.64	1.86	54.2	4758	92.6
Tripura	9.8	11.05	21.11	58.04	1.70	13.9	1080	67.5
Uttar Pradesh	6.76	10.56	12.97	69.71	2.86	17.3	781	58.7
Uttarakhand	8.25	1.56	7.88	82.32	2.12	27.4	306	35.0
West Bengal	10.45	14.47	18.98	56.1	1.83	29.3	1455	77.0
India	12.30	7.49	19.79	60.42	2.25	36.0	1821	79.1

States with sample size less than 50 were not included

Mean OOP is estimated at constant price with base year of 2016

The state differentials in mean OOP and its share to the total expenditure on female sterilization were high. The OOP in states such as Madhya Pradesh (−₹173) and Rajasthan (−₹143) with high utilization of public health facilities for female sterilization had negative OOP. The negative OOP is due to the fact that the compensation received is higher than the money spent. Besides, in these two states, over 90% of the acceptors availed themselves of sterilization at public health centers. The OOP for female sterilization was highest in Nagaland (₹8423) followed by Manipur (₹7390) and Kerala (₹6742). The share of OOP was more than 90% in Daman and Diu, Manipur, Kerala, Nagaland, Meghalaya, Mizoram and Telangana. It was comparatively less in states where the utilization of female sterilization from the public facility was more than 90%.

### Socioeconomic and demographic variations of OOP on female sterilization

Table 2 presents the differentials in mean OOP and its share in the total expenditure for female sterilization by selected socio-economic and demographic characteristics in India, Uttar Pradesh and Odisha at constant prices during 2010–14. The states of Uttar Pradesh and Odisha are selected as illustrations to reflect the variations in OOP across the states, as the use of sterilization from public health centers is high in Odisha and from private health centers is high in Uttar Pradesh. The OOP for female sterilization was positively associated with educational attainment. It was about eight times higher for females with higher education compared to uneducated women in India. The share of OOP in the total expenditure was more than double for those women with above secondary level education. The OOP was highest among ‘others’ in the caste category, higher in urban than rural areas and also varied with the respondent’s perception on the quality of care of female sterilization services. Those women who stated that the quality of care was good in India and Uttar Pradesh had incurred a higher OOP. The mean OOP in Odisha varied from ₹640 to ₹275 among those stating that the quality of care was ‘not good’ and those stating that it was ‘good’ respectively. The OOP was positively associated with the wealth quintile suggesting that the burden was higher among those who had the ability to pay more. The mean OOP varied between ₹5181 and ₹201among women belonging to the richest wealth quintile and the poorest wealth quintile respectively in India.

**Table 2:** Mean OOP and its share to the total expenditure on female sterilization by background characteristics at constant prices (₹), India, 2010–2014

Variables	India		Uttar Pradesh		Odisha	
	Mean OOP (₹)	OOP as a share of total expenditure	Mean OOP (₹)	OOP as a share of total expenditure	Mean OOP (₹)	OOP as a share of total expenditure
Respondent’s Age (years)						
< 25	1523	73.6	1182	62.3	149	17.6
25–34	1714	76.6	606	49.5	199	22.3
35–49	2100	82.5	1005	66.1	475	46.6
Education Level						
No education	535	47.4	410	41.1	− 279	−73.6
Primary	887	60.9	476	41.7	46	6.8
Secondary	1737	77.8	941	62.4	693	51.0
Higher	4034	91.5	2748	84.8	1540	73.0
Religion						
Hindu	1605	75.4	663	52.8	313	32.7
Muslim	3324	89.5	2578	85.1	116	21.0
Others	2199	82.7	282	44.8	− 332	−88.5
Caste						
SC	841	60.0	275	31.5	172	20.8
ST	372	37.2	− 179	−37.2	− 342	− 109.2
OBC	1993	79.5	754	56.0	575	47.2
Others	3070	88.9	2084	80.2	961	61.4
Place of Residence						
Urban	3254	89.3	2591	84.6	1401	70.8
Rural	1191	68.1	449	42.7	137	17.3
No. of Surviving Children						
< 2	2537	84.3	2010	78.8	576	46.7
3	1140	68.3	679	52.9	56	8.1
4+	554	48.8	302	34.0	− 249	−64.4
Quality of care						
Good	1800	78.0	803	57.9	275	29.9
Not Good	1078	67.0	405	40.5	640	51.3
Wealth Index						
Poorest	201	24.1	22	3.4	−198	−42.4
Poorer	572	49.5	290	32.5	−57	−9.2
Middle	1350	72.0	1069	65.2	443	42.8
Richer	2943	87.3	1843	77.8	1444	70.6
Richest	5181	94.7	4732	91.9	5651	92.2
Type of facility						
Public Health Center	− 313	− 103.6	− 450	− 216.3	−167	−33.6
Private Health Center	10,766	99.5	10,162	99.7	9566	97.0

### Correlates of OOP and predicted OOP on female sterilization in India

Table 3 shows the result of a two-part regression model and predicted expenditure on sterilization in India. The results of the logit regression show that the likelihood of incurring OOP on sterilization was positively associated with age, education level, economic status of the woman and sterilization by type of facility. For instance, women aged 35–49 were 44% more likely to incur OOP on sterilization compared to woman aged less than 25 years. The likelihood of incurring OOP on sterilization was 51% higher among women with higher education compared to women with no education. Similarly, the likelihood of incurring OOP was higher among women belonging to the richest wealth quintile compared to women from the poorest quintile. Further, a woman utilizing sterilization from a private health facility was significantly more likely to incur OOP compared to those utilizing sterilization from a public health center.

**Table 3:** Results of the two-part regression model and predicted OOP on female sterilization in India, 2015–16

Background Characteristics	*β* (logit)	Confidence Interval	*β* (OLS)	Confidence Interval	Predicted Mean Cost of OOP (₹)
Respondent’s Age (years)					
Less than 25	1		1		2307
25–34	0.149**	(0.032, 0.265)	0.094	(−0.037, 0.236)	2698
35–49	0.444***	(0.302, 0.586)	0.236***	(0.073, 0.399)	3737
Education Level					
No education	1		1		1671
Primary	0.145**	(0.026, 0.265)	0.056	(−0.076, 0.188)	2539
Secondary	0.225***	(0.115, 0.336)	0.051	(−0.078, 0.181)	3562
Higher Secondary and Above	0.513***	(0.392, 0.634)	0.077	(−0.071, 0.225)	6984
Religion					
Hindu	1		1		3130
Muslim	0.192***	(0.046, 0.339)	0.1	(− 0.058, 0.257)	5336
Others	0.087	(−0.119, 0.292)	− 0.044	(− 0.269, 0.181)	4100
Caste					
SC	1		1		1859
ST	−0.178**	(− 0.340, − 0.017)	0.073	(− 0.109, 0.254)	1199
OBC	0.156***	(0.060, 0.251)	0.121**	(0.010, 0.232)	3730
Others	0.409***	(0.282, 0.537)	0.184***	(0.045, 0.232)	4950
Place of Residence					
Urban	1		1		5364
Rural	−0.073	(−0.171, 0.024)	0.057	(−0.043, 0.156)	2328
Number of Surviving Children					
< 2	1		1		4595
3	−0.195***	(− 0.292, − 0.097)	−0.159***	(− 0.271, − 0.047)	2380
4+	− 0.289***	(− 0.402, − 0.175)	−0.229***	(− 0.362, − 0.096)	1458
Quality of Care					
Good	1		1		3388
Not Good	0.146	(−0.042, 0.333)	−0.303***	(− 0.527, − 0.078)	2033
Wealth Index					
Poorest	1		1		676
Poorer	0.04	(−0.070, 0.150)	0.248***	(0.104, 0.391)	1099
Middle	0.289***	(0.168, 0.411)	0.420***	(0.277, 0.563)	2088
Richer	0.735***	(0.598, 0.872)	0.680***	(0.520, 0.840)	4497
Richest	1.416***	(1.249, 1.584)	0.748***	(0.565, 0.932)	7670
Type of facility					
Public Health Center	1		1		669
Private Health Center	5.31***	(5.15, 5.48)	1.63***	(1.52, 1.73)	6428

Note: ***p < 0.01, **p < 0.05, *p < 0.10 (indicates statistically significant)

For the second part, the log transformation of woman who incurred any OOP was used as a dependent variable. The probability of incurring any OOP on sterilization was higher among older women, belonging to the richest wealth quintile and availing themselves of sterilization from a private health center. The probability of incurring any OOP on sterilization was 23.6% higher among women aged 35–49 years compared to women aged less than 25 years. Further, the probability of incurring any OOP was 74.8% higher among women belonging to the richest wealth quintile compared to women from the poorest quintile. Similarly, the probability of incurring any OOP among women using a private health center for sterilization was about twice higher compared to women using a public health center.

The predicted mean expenditure on sterilization was 62% higher for women aged 35–49 (₹3737) compared to women aged less than 25 years (₹2307). Similarly, the predicted mean was four times for women with higher education (₹6984) compared to women with no education (₹1671). On an average, the predicted mean expenditure was eleven times higher for a woman belonging to the richest wealth quintile (₹7670) compared to a woman from the poorest quintile (₹676). The OOP was almost ten times higher for a woman utilizing sterilization from a private health center (₹6428) compared to a public health center (₹669).

For illustrating the state patterns, we have estimated the coefficients and OOP for Uttar Pradesh and Odisha. Table S3 presents the results of the two-part regression model to identify the correlates of incurred OOP and present the adjusted predicted mean OOP. In general, similar patterns were observed for Odisha and Uttar Pradesh. The predicted OOP for women aged 35+ years in Odisha was three times higher than that for women below 25 years. The OOP on sterilization increases with the educational level of the women in both the states. On an average, the mean OOP on female sterilization was about three times (₹6112) and ten times (₹5248) more for women with higher secondary education and above, compared to women with no education in Uttar Pradesh and Odisha respectively.

## Discussion

Fertility reduction in India is largely attributed to increased use of female sterilization and increase in female age at marriage. Female sterilization continued to be the most preferred and dominant method of limiting family size across socioeconomic groups in India. It is popular among the poor and the less educated. India’s family welfare programme provides not only free family planning services but compensation is also paid to acceptors towards wage loss, transportation to and from the facility, expenses of food, child care during hospital stay and laboratory fees for any tests. Besides, the central and state governments, the private sector and non-governmental organizations have been working towards promoting voluntary family planning services including female sterilization. Despite this, the regional patterns in the use of modern contraceptive methods are strikingly low in the poorer states of Bihar, Odisha, Jharkhand and Uttar Pradesh and the unmet need for limiting and spacing is higher in these states. Besides, the quality of family planning services remains a concern. The national, central and local governments and international donors continue to invest heavily in family planning programmes in India to achieve the desired family size, meeting the unmet need and providing family planning services at no-cost at public health centers, but the household does not necessarily receive free services. Though the demographic, health and social benefits of family planning programmes in India have been examined periodically, few studies focus on the economic benefits of family planning investments. Periodic assessments of economic benefits are necessary for evidence based policy and programmes. In this context, this is the first ever comprehensive study that examines the expenditure, compensation received and OOP for female sterilization in India. We have used the unit data of the recently released NFHS 4 that provided information on amount spent and compensation received on sterilization. The salient findings are:

- **First**, public health centers are the most preferred type of facility for undergoing female sterilization in India. Over 85% acceptors of female sterilization did not pay for availing themselves of the services and those are largely from public health centers. Similarly, a large proportion of sterilization acceptors from public health centers received compensation and the trend remains the same over time. Over 90% of acceptors from private health centers paid for the services. Despite this, the utilization of female sterilization in private health centers has increased over time.
- **Second**, the state pattern in the use of sterilization (among major states) by type of facility suggests that the use of sterilization in private health centers was highest in Kerala (49%) followed by Karnataka and Telangana. On the other hand, the use of female sterilization in public health centers was highest in Chandigarh followed by Haryana and Odisha.
- **Third**, the amount spent and compensation received and the OOP for female sterilisation varied across states in India. At the national level, about 12% of the women neither paid nor received compensation, 8% paid and received compensation, 20% paid for female sterilization but did not receive any compensation, while about 60% of the women did not pay for the service but received compensation. Among the major states, in Kerala, about 42% of the women paid but did not receive any compensation followed by Manipur and Nagaland (39%), in contrast to Madhya Pradesh and Himachal Pradesh where it was around 4%. In Himachal Pradesh, 88% of the women did not pay but received compensation followed by Madhya Pradesh (84%) and the least was in Nagaland (23%) and Manipur (30%).
- **Fourth**, the OOP as a share of the total amount spent on female sterilization in India varies between 70 and 79%, lower in public health centers and higher in private health centers. The state variations in OOP on female sterilization were prominent. A higher proportion of women from the economically developed states of Kerala, Karnataka, and Delhi and from the economically poorer states of Meghalaya and Bihar incurred OOP for undergoing female sterilization.
- **Fifth**, the amount of OOP for female sterilization varied widely across the public and private centers across the states of India. The total expenditure at the private health centers was many times higher than that in pubic health centers. The OOP on female sterilization was positively and significantly associated with educational attainment, share of urban population, economic well-being of households and better quality of care. This suggests that women belonging to households with better socioeconomic conditions are paying for services from private health centers and for better quality of care. However, the multivariate analyses suggested that OOP was also higher among women from scheduled castes.

We provide some explanations in support of the results. Public health centers continue to be the major provider of female sterilization services in most states in India. The use of female sterilization from private health centers has been increasing over time possibly due to convenience, efficiency, quality of care and improved standard of living of the population. Previous studies suggest that about 10% of the sterilization acceptors from public health centers paid for services in 2015. The mean expenditure for carrying out female sterilization in private facilities was around ₹3400 [68]. About half of the women in Kerala are opting for sterilization in private health centers, which is suggestive of preferences and ability to pay for the services. The higher proportion of female sterilization acceptors in Bihar suggests the lack of facilities in public health centers. Similarly, lack of proper infrastructure and lack of access to public health facilities may hinder the utilization of sterilization among women in the marginalized sections of society. Reasons reported for the low use of services in public health centers “the waiting time” and “disrespectful behaviour” [69]. Further, under the Public Private Partnerships (PPPs), more private facilities have been accredited by the government to increase the provider base for family planning services. However, our findings suggest that the proportion of women using private health centers and receiving compensation is very low (less than 5%).

The OOP, in general, is associated with the ability to pay for female sterilization services with the exception of Bihar, Nagaland, Meghalaya and Mizoram. The proportion of women who paid but did not receive compensation was higher in many economically backward states such as Nagaland, Meghalaya, Mizoram and Bihar. The share of women who opted for female sterilization was much lower in these states with a greater prevalence of high OOP and a higher share of acceptance of female sterilization from private facilities compared to other states. While female sterilization was less than 10% for Nagaland, Manipur and Meghalaya, it was around 20% for Bihar, nearly 50% of the estimate at the national level. Also, the share of women who had not paid but received compensation was higher in states such as Haryana where there was higher utilization of services from public health centers. The reduction in the ratio of total expenditure on female sterilization from private and public health centers could be attributed to the public private partnership (PPP) programmes through which accredited private health centers also provide compensation to sterilization acceptors.

These findings have implications for equity-driven interventions on sterilization in India.

**First**, the public and private differentials in OOP on sterilization are large across states and socio-economic groups. Given the increasing use of services from private health centers, state interventions to regulate the price in private health center are called for. This, in-turn would reduce the catastrophic health spending and distress financing on sterilization. For example, the high OOP even in many of the poorer states such as Mizoram, Meghalaya and Nagaland warrant larger investment on family planning services.

**Second,** the high OOP among socially disadvantaged groups such as Scheduled Castes and Scheduled Tribes needs programmatic attention. While provisioning of such services in public health centers are free or subsidised, private health centers does not provide subsidised services to any group of population. In such cases, the public-private partnership may be strengthened to reduce the OOP burden for disadvantaged groups.

**Third,** public health centers continue to be the largest providers of sterilization services in India. Thus, improving the quality of female sterilization services in and across states is important for improving women’s health.

**Fourth**, the compensation provided for female sterilization should exclude women from higher socioeconomic households. Although, the compensation is set low enough so that people do not access sterilization because they need the compensation, and it implies no coercion in sterilization, the compensation received for female sterilization should be revised for certain lagging sections of the population.

Although the findings offer important insights into the economics of female sterilization within family planning programmes, the results must be interpreted in the light of certain study limitations. These include underestimation of costs as it does not include indirect costs associated with female sterilization such as loss of wages, transport to and from facility, expenses for food and child care during hospital stay, and laboratory fees for related tests. The questions on quality of care were not exhaustive as they were based on the perception of the receptors, and did not capture all the essential dimensions of quality of care.

## Conclusion

Based on the results, this study concludes that investment in family planning in public health centers should continue as these services are largely availed by the poor, less educated and marginalized population. Family planning programmes could benefit from an equity- driven focus and there is an urgent need to regulate the private sector on cost and quality of services. Public and private sector investments as per public private partnerships (PPP) should increase coverage for accessibility of voluntary family planning services and to achieve the desired family size. Women in the poorer and high fertility states such as Bihar and north-eastern states such as Manipur and Meghalaya needs further attention as OOP is high in those states.

## Supplementary information

**Additional file 1:** **Table S1.** Percent distribution of women who paid for female sterilization and received compensation by year in India at current prices (in INR), 1990–2014. **Table S2.** Percent distribution of women who paid for female sterilization and received compensation by year and type of facility in India at current prices (in Indian Rupees), 1990–2014. **Table S3.** Results of Two-part model and predicted OOP payment on female sterilization in Uttar Pradesh and Odisha, 2015–16

## Data Availability

The dataset used for the current study is available in DHS repository. Available from: [https://dhsprogram.com/data/dataset/India_Standard-DHS_2015.cfm?flag=0].

## Abbreviations

CPI: Consumer Price Index
DK: Do Not Know
IUD: Intrauterine Device
MCH: Maternal and Child Health
NFHS: National Family Health Survey
NGO: Non-Governmental Organisation
NHM: National Health Mission
OBC: Other Backward Classes
OOP: Out -of -Pocket Payment
PPP: Public Private Partnership
RBI: Reserve Bank of India
RCH: Reproductive and Child Health
SC: Scheduled Caste
ST: Scheduled Tribe
TFR: Total Fertility Rate

## Footnotes

1 High Focus States: UP, Bihar, Chhattisgarh, Jharkhand, Odisha, MP, Rajasthan, Gujarat, Uttarakhand, Assam, Haryana.

2 The public centers include Government/ municipal hospitals, sub-centers, public health centers, community health centers, district hospitals and camps. The private centers include private hospitals, private clinic, and private mobile clinic. Non-governmental organisations (NGOs), trust hospitals or clinics are classified as ‘others’.
