# Supplementary material for: Pattern and correlates of out-of-pocket payment (OOP) on female sterilization in India, 1990–2014
Source: BMC Womens Health. 2020 Jan 22;20:13. doi: 10.1186/s12905-020-0884-1 (PMC6977276; doi:10.1186/s12905-020-0884-1)
Supplement: Supplementary file 1 — Additional file 1: Table S1. Percent distribution of women who paid for female sterilization and received compensation by year in India at current prices (in INR), 1990–2014. Table S2. Percent distribution of women who paid for female sterilization and received compensation by year and type of facility in India at current prices (in Indian Rupees), 1990–2014. Table S3. Results of Two-part model and predicted OOP payment on female sterilization in Uttar Pradesh and Odisha, 2015–16 [file 12905_2020_884_MOESM1_ESM.docx]

Table S1: Percent distribution of women who paid for female sterilization and received compensation by year in India at current prices (in INR), 1990-2014

| **Year** | **Neither paid nor received compensation** | **Paid and received compensation** | **Paid and did not receive compensation** | **Did not pay but received compensation** | **Total Expenditure** | **OOP payment as a share of total expenditure** | **N** |
| --- | --- | --- | --- | --- | --- | --- | --- |
| 1990 | 24.0 | 4.4 | 10.2 | 61.5 | 661 | 69.9 | 3,623 |
| 1991 | 22.4 | 3.9 | 10.5 | 63.2 | 691 | 70.4 | 1,582 |
| 1992 | 21.9 | 4.4 | 10.2 | 63.5 | 827 | 74.4 | 2,153 |
| 1993 | 21.5 | 3.0 | 12.5 | 62.9 | 931 | 78.2 | 2,757 |
| 1994 | 23.1 | 4.3 | 12.2 | 60.4 | 742 | 71.2 | 3,354 |
| 1995 | 23.3 | 4.8 | 13.4 | 58.5 | 981 | 78.9 | 4,057 |
| 1996 | 24.8 | 4.4 | 13.2 | 57.5 | 1055 | 80.0 | 4,376 |
| 1997 | 25.0 | 4.7 | 13.5 | 56.8 | 903 | 76.7 | 4,847 |
| 1998 | 25.4 | 4..0 | 14.0 | 56.5 | 905 | 76.9 | 5,343 |
| 1999 | 26.0 | 4.2 | 15.5 | 54.4 | 1113 | 81.1 | 6,002 |
| 2000 | 25.9 | 4.0 | 15.6 | 54.5 | 1084 | 80.1 | 7,424 |
| 2001 | 26.5 | 4.2 | 15.6 | 53.6 | 1086 | 79.0 | 6,502 |
| 2002 | 25.5 | 4.0 | 17.0 | 53.4 | 1183 | 81.4 | 7,086 |
| 2003 | 24.0 | 5.2 | 16.4 | 54.3 | 1111 | 79.1 | 7,592 |
| 2004 | 22.0 | 4.7 | 16.8 | 56.6 | 1256 | 80.0 | 7,740 |
| 2005 | 20.7 | 4.9 | 18.7 | 55.8 | 1346 | 81.4 | 8,401 |
| 2006 | 19.6 | 4.7 | 19.2 | 56.6 | 1368 | 79.6 | 7,983 |
| 2007 | 16.0 | 5.6 | 18.4 | 60.0 | 1549 | 77.9 | 8,073 |
| 2008 | 14.1 | 6.5 | 18.0 | 61.5 | 1442 | 73.3 | 8,840 |
| 2009 | 15.1 | 6.2 | 17.0 | 61.6 | 1519 | 74.2 | 9,391 |
| 2010 | 13.1 | 6.8 | 19.5 | 60.6 | 1809 | 77.7 | 9,186 |
| 2011 | 13.0 | 7.1 | 18.4 | 61.5 | 1878 | 78.0 | 9,072 |
| 2012 | 12.5 | 7.8 | 19.9 | 59.9 | 2248 | 81.4 | 8,174 |
| 2013 | 12.0 | 7.6 | 20.4 | 60.1 | 2232 | 80.4 | 8,131 |
| 2014 | 10.8 | 8.3 | 20.9 | 60.0 | 2320 | 78.8 | 7,791 |

Table S2: Percent distribution of women who paid for female sterilization and received compensation by year and type of facility in India at current prices (in Indian Rupees), 1990-2014

| **Year** | **Public** | | | | | **Private** | | | | | **Ratio of total expenditure (Private/Public)** |
| --- | --- | --- | --- | --- | --- | --- | --- | --- | --- | --- | --- |
|  | **Neither paid nor received** | **Paid and received compensation** | **Paid and did not receive compensation** | **Did not pay but received compensation** | **Total expenditure** | **Neither paid nor received** | **Paid and received compensation** | **Paid and did not receive compensation** | **Did not pay but received compensation** | **Total expenditure** |  |
| 1990 | 25.9 | 4.1 | 1.9 | 68.1 | 97 | 7.3 | 6.6 | 80.5 | 5.6 | 5406 | 55.8 |
| 1991 | 23.9 | 3.6 | 2.4 | 70.1 | 124 | 9.0 | 6.2 | 78.2 | 6.6 | 5345 | 43.1 |
| 1992 | 23.8 | 4.1 | 1.4 | 70.7 | 137 | 6.3 | 6.3 | 79.7 | 7.7 | 6193 | 45.1 |
| 1993 | 23.8 | 2.8 | 2.5 | 70.9 | 84 | 5.4 | 3.9 | 83.2 | 7.4 | 6806 | 80.6 |
| 1994 | 25.1 | 4.3 | 2.2 | 68.5 | 101 | 9.0 | 4.8 | 80.7 | 5.4 | 5061 | 50.3 |
| 1995 | 26.0 | 4.7 | 2.5 | 66.8 | 129 | 5.8 | 5.1 | 84.6 | 4.5 | 6470 | 50.2 |
| 1996 | 27.7 | 4.1 | 2.3 | 65.9 | 123 | 5.7 | 5.9 | 84.3 | 4.2 | 6978 | 56.7 |
| 1997 | 28.2 | 4.4 | 3.1 | 64.4 | 149 | 4.5 | 6.3 | 82.7 | 6.5 | 5875 | 39.5 |
| 1998 | 28.7 | 3.8 | 3.0 | 64.5 | 118 | 5.2 | 5.7 | 81.0 | 8.2 | 5921 | 50.0 |
| 1999 | 29.5 | 4.0 | 3.8 | 62.7 | 149 | 5.2 | 5.2 | 85.5 | 4.2 | 6612 | 44.5 |
| 2000 | 29.3 | 4.0 | 4.4 | 62.4 | 183 | 3.7 | 4.5 | 88.5 | 3.3 | 6894 | 37.7 |
| 2001 | 29.5 | 4.1 | 4.9 | 61.5 | 164 | 5.8 | 5.1 | 85.3 | 3.8 | 7043 | 42.9 |
| 2002 | 29.1 | 4.1 | 4.4 | 62.5 | 195 | 5.9 | 3.6 | 86.0 | 4.6 | 6503 | 33.3 |
| 2003 | 27.3 | 5.2 | 3.8 | 63.7 | 138 | 5.9 | 5.0 | 86.2 | 2.9 | 6253 | 45.2 |
| 2004 | 25.1 | 5.0 | 3.7 | 66.2 | 174 | 4.1 | 2.9 | 90.0 | 3.0 | 7237 | 41.7 |
| 2005 | 24.1 | 5.1 | 4.4 | 66.4 | 195 | 3.9 | 3.3 | 90.3 | 2.5 | 7075 | 36.3 |
| 2006 | 23.1 | 4.6 | 3.9 | 68.4 | 197 | 3.3 | 4.9 | 89.2 | 2.5 | 6684 | 33.9 |
| 2007 | 18.3 | 5.9 | 3.8 | 72.0 | 192 | 4.9 | 4.3 | 87.0 | 3.7 | 7823 | 40.7 |
| 2008 | 15.8 | 6.9 | 3.7 | 73.7 | 204 | 5.8 | 4.5 | 86.3 | 3.5 | 7308 | 35.8 |
| 2009 | 16.9 | 6.7 | 3.4 | 73.1 | 243 | 5.9 | 3.9 | 87.6 | 2.6 | 8098 | 33.3 |
| 2010 | 15.2 | 7.5 | 3.2 | 74.1 | 252 | 3.8 | 3.9 | 90.6 | 1.7 | 8581 | 34.0 |
| 2011 | 14.6 | 7.9 | 3.4 | 74.1 | 249 | 5.1 | 3.6 | 88.7 | 2.7 | 9429 | 37.8 |
| 2012 | 14.4 | 8.5 | 3.4 | 73.7 | 342 | 4.3 | 4.9 | 88.8 | 1.9 | 10277 | 30.1 |
| 2013 | 14.0 | 8.6 | 3.0 | 74.5 | 239 | 4.2 | 3.3 | 90.0 | 2.4 | 10215 | 42.7 |
| 2014 | 12.4 | 9.7 | 3.5 | 74.5 | 306 | 4.7 | 2.9 | 90.5 | 2.0 | 10304 | 33.7 |

Table S3: Results of Two-part model and predicted OOP payment on female sterilization in Uttar Pradesh and Odisha, 2015-16

| **Background Characteristics** | **Uttar Pradesh** | | | | | **Odisha** | | | | |
| --- | --- | --- | --- | --- | --- | --- | --- | --- | --- | --- |
|  | ***β* (logit)** | **Confidence Interval** | ***β* (OLS)** | **Confidence Interval** | **Predicted mean cost of OOP** | ***β* (logit)** | **Confidence Interval** | ***β* (OLS)** | **Confidence Interval** | **Predicted mean cost of OOP** |
|  |  |  |  |  |  |  |  |  |  |  |
| **Respondent's Age (years)** |  |  |  |  |  |  |  |  |  |  |
| Less than 25 | 1 |  | 1 |  | 1795 | 1 |  | 1 |  | 746 |
| 25-34 | -0.139 | (-0.580, 0.302) | -0.15 | (-1.109, 0.810) | 1340 | -0.464* | (-0.993, 0.066) | 0.544 | (-0.139, 1.227) | 1116 |
| 35-49 | 0.192 | (-0.285, 0.669) | 0.24 | (-0.758, 1.238) | 2755 | -0.640** | (-1.217, -0.063) | 1.042*** | (0.332, 1.762) | 2122 |
| **Education Level** |  |  |  |  |  |  |  |  |  |  |
| No education | 1 |  | 1 |  | 1450 | 1 |  | 1 |  | 571 |
| Primary | -0.112 | (-0.429, 0.206) | 0.164 | (-0.255, 0.58.) | 2297 | 0.312 | (-0.092, 0.716) | 0.208 | (-0.209, 0.625) | 1327 |
| Secondary | 0.047 | (-0.247, 0.340) | -0.056 | (-0.497, 0.385) | 2593 | 0.13 | (-0.251, 0.511) | 0.560*** | (0.153, 0.966) | 2972 |
| Higher | 0.257 | (-0.086, 0.599) | 0.117 | (-0.394, 0.629) | 6112 | 0.197 | (-0.331, 0.725) | 0.137 | (-0.538, 0.813) | 5248 |
| **Religion** |  |  |  |  |  |  |  |  |  |  |
| Hindu | 1 |  | 1 |  | 2137 | 1 |  | 1 |  | 1844 |
| Muslims | 0.773*** | (0.396, 1.150) | 0.355 | (-0.101, 0.812) | 5753 | -0.128 | (-1.883, 1.628) | -1.711*** | (-2.344, -1.078) | 464 |
| Others | 0.581 | (-1.297, 2.459) | -0.278 | (-1.419, 0.862) | 4512 | -0.383 | (-0.941, 0.175) | -0.275 | (-0.650, 0.099) | 583 |
| **Caste** |  |  |  |  |  |  |  |  |  |  |
| SC | 1 |  | 1 |  | 1187 | 1 |  | 1 |  | 1080 |
| ST | -0.526 | (-1.409, 0.358) | -0.078 | (-0.348, 0.431) | 637 | -0.573** | (-0.984, -0.162) | -0.258 | (-0.714, 0.197) | 351 |
| OBC | 0.163 | (-0.088, 0.414) | 0.042 | (-0.536, 0.543) | 2194 | -0.083 | (-0.432, 0.267) | 0.289 | (-0.070, 0.647) | 2176 |
| Others | 0.258 | (-0.094, 0.610) | 0.004 | (-0.527, 0.214) | 4199 | -0.012 | (-0.476, 0.452) | 0.085 | (-0.397, 0.566) | 3745 |
| **Place of residence** |  |  |  |  |  |  |  |  |  |  |
| Urban | 1 |  | 1 |  | 5238 | 1 |  | 1 |  | 5652 |
| Rural | -0.259* | (-0.534, -0.016) | 0.037 | (-0.380, 0.454) | 1527 | -0.125 | (-0.537, 0.286) | -0.254 | (-0.664, 0.157) | 1073 |
| **Number of surviving children** |  |  |  |  |  |  |  |  |  |  |
| Less than equal to 2 | 1 |  | 1 |  | 4520 | 1 |  | 1 |  | 2372 |
| 3 | -0.507*** | (-0.772, -0.242) | -0.156 | (-0.527, 0.214) | 2182 | 0.008 | (-0.332, 0.347) | -0.03 | (-0.362, 0.302) | 1548 |
| 4+ | -0.720*** | (-1.025, -0.415) | -0.219 | (-0.639, 0.201) | 1490 | -0.007 | (-0.435, 0.421) | -0.368 | (-0.805, 0.069) | 769 |
| **Quality of care** |  |  |  |  |  |  |  |  |  |  |
| Good | **1** |  | 1 |  | 2384 | 1 |  | 1 |  | 1780 |
| Not Good | 0.376* | (-0.033, 0.784) | -0.648** | (-1.133, -0.162) | 1442 | 0.671 | (-0.006, 1.348) | -0.221 | (-0.808, 0.366) | 2268 |
| **Wealth Index** |  |  |  |  |  |  |  |  |  |  |
| Poorest | **1** |  | 1 |  | 597 | 1 |  | 1 |  | 538 |
| Poorer | 0.134 | (-0.149, 0.418) | 0.033 | (-0.430, 0.497) | 796 | 0.295* | (-0.045, 0.635) | -0.263 | (-0.661, 0.135) | 713 |
| Middle | 0.349** | (0.046, 0.652) | 0.673** | (0.223, 1.124) | 2044 | 0.474** | (0.065, 0.884) | -0.446* | (-0.962, 0.069) | 833 |
| Richer | 0.440** | (0.087, 0.793) | 0.994*** | (0.500, 1.488) | 3553 | 0.584** | (0.045, 1.123) | 0.679* | (-0.067, 1.424) | 3384 |
| Richest | 0.895 | (0.467, 1.323) | 1.236*** | (0.590, 1.883) | 7867 | 1.030*** | (0.269, 1.791) | 1.731*** | (1.068, 2.394) | 14772 |
| **Type of facility** |  |  |  |  |  |  |  |  |  |  |
| Public Health Centre | 1 |  | 1 |  | 143 | 1 |  | 1 |  | 484 |
| Private Health Centre | 4.96*** | (4.53, 5.38) | 1.94*** | (1.62, 2.26) | 14370 | 3.00*** | (2.39, 3.61) | 1.39 | (0.99, 1.78) | 19540 |

****p<0.01, **p<0.05, *p<0.10*
